# Computed tomography imaging‐based observation of the aggressive growth of angiosarcoma: a case study

**DOI:** 10.1002/rcr2.479

**Published:** 2019-08-26

**Authors:** Sousuke Kubo, Nobuaki Kobayashi, Ayami Kaneko, Hiroko Aiko, Makoto Kudo, Takeshi Kaneko

**Affiliations:** ^1^ Department of Pulmonology Yokohama City University Graduate School of Medicine Yokohama Japan; ^2^ Department of Pulmonology Yokohama City University Medical Center Yokohama Japan

**Keywords:** Angiosarcoma, chronic empyema, chronic inflammation, malignant chest wall tumour, primary chest wall tumour

## Abstract

An 82‐year‐old man with chronic tuberculous empyema visited our hospital for an annual computed tomography (CT) scan. No differences were noted between the CT scan at presentation and a scan performed a year previously in August 2017. He began experiencing right chest, epigastrium, and back pain since the end of October 2017. A CT scan taken in November of 2017 to evaluate the pain in his right chest, epigastrium, and back showed an irregular thickening of the pleura adjacent to the empyema and an abnormal right seventh costal mass infiltrating the vertebral body. CT‐guided needle biopsy of the mass showed angiosarcoma. Positron emission tomography/CT revealed multiple metastases in his bones and liver. Chemotherapy was not recommended owing to his poor performance status, which was related to angiosarcoma. Therefore, he was offered palliative radiotherapy for the metastasis to the vertebral body.

## Introduction

Primary malignant chest wall tumour is a rare disease caused by chronic inflammation, including chronic empyema 1. Unlike pyothorax‐associated lymphoma and squamous cell lung carcinoma, angiosarcoma is a less common primary malignant chest wall tumour, and accounts for approximately 4% of cases. Angiosarcoma is known for its rapid progression, often conferring poor prognosis. Here, we describe a case of angiosarcoma with aggressive growth observed on chest computed tomography (CT) at three months from diagnosis and provide a comprehensive review of literature.

## Case Report

**Figure 1:**
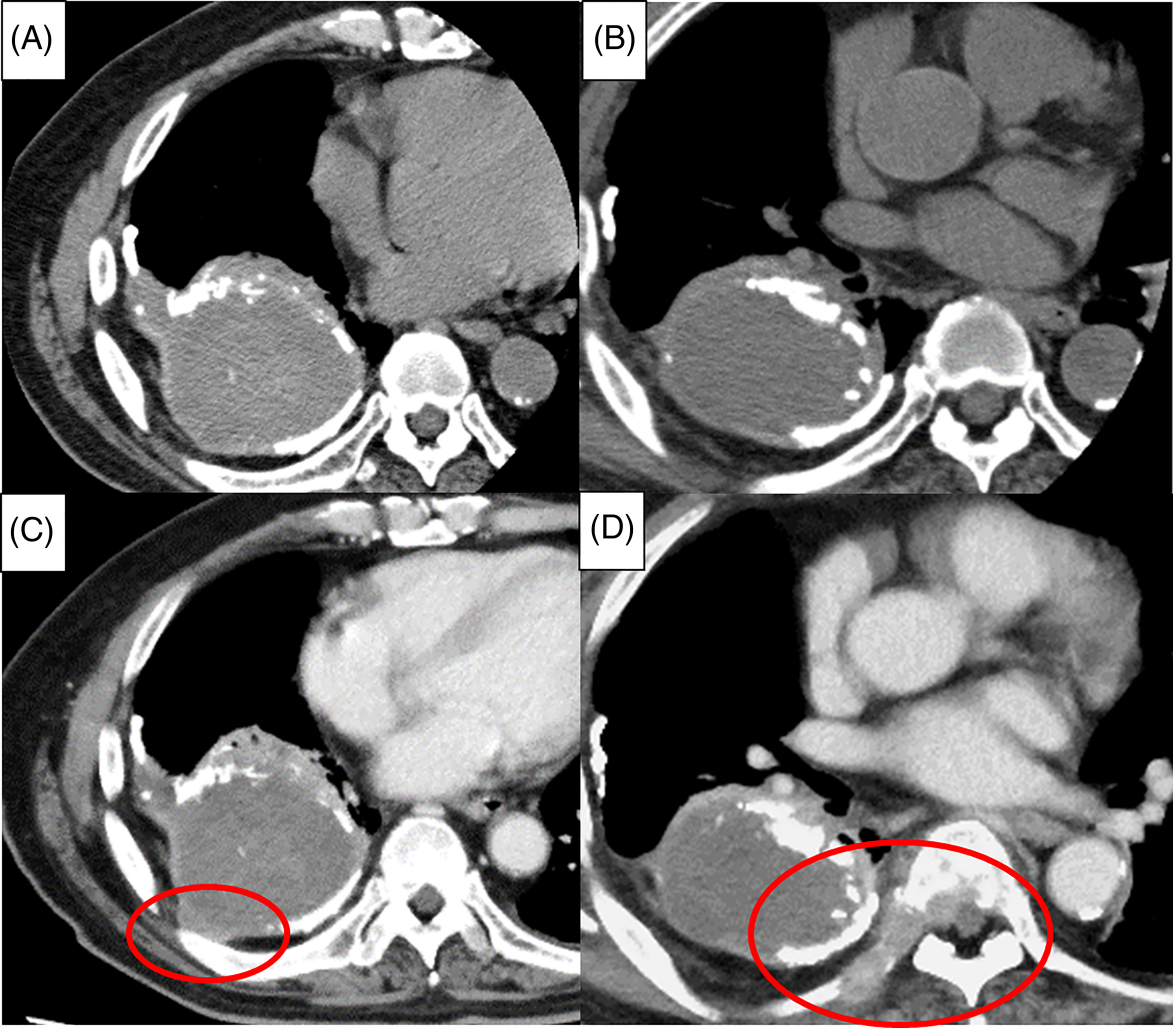
Computed tomography (CT) scan in August 2017. (A, B) Although chronic empyema is also seen in the previous scan, there is no evidence of malignancy. (C, D) A contrast CT scan in mid‐November 2017 revealing the irregular protruding posterior pleural thickening adjacent to the chronic empyema, and an abnormal right seventh costal mass infiltrating the vertebral body.

**Figure 2:**
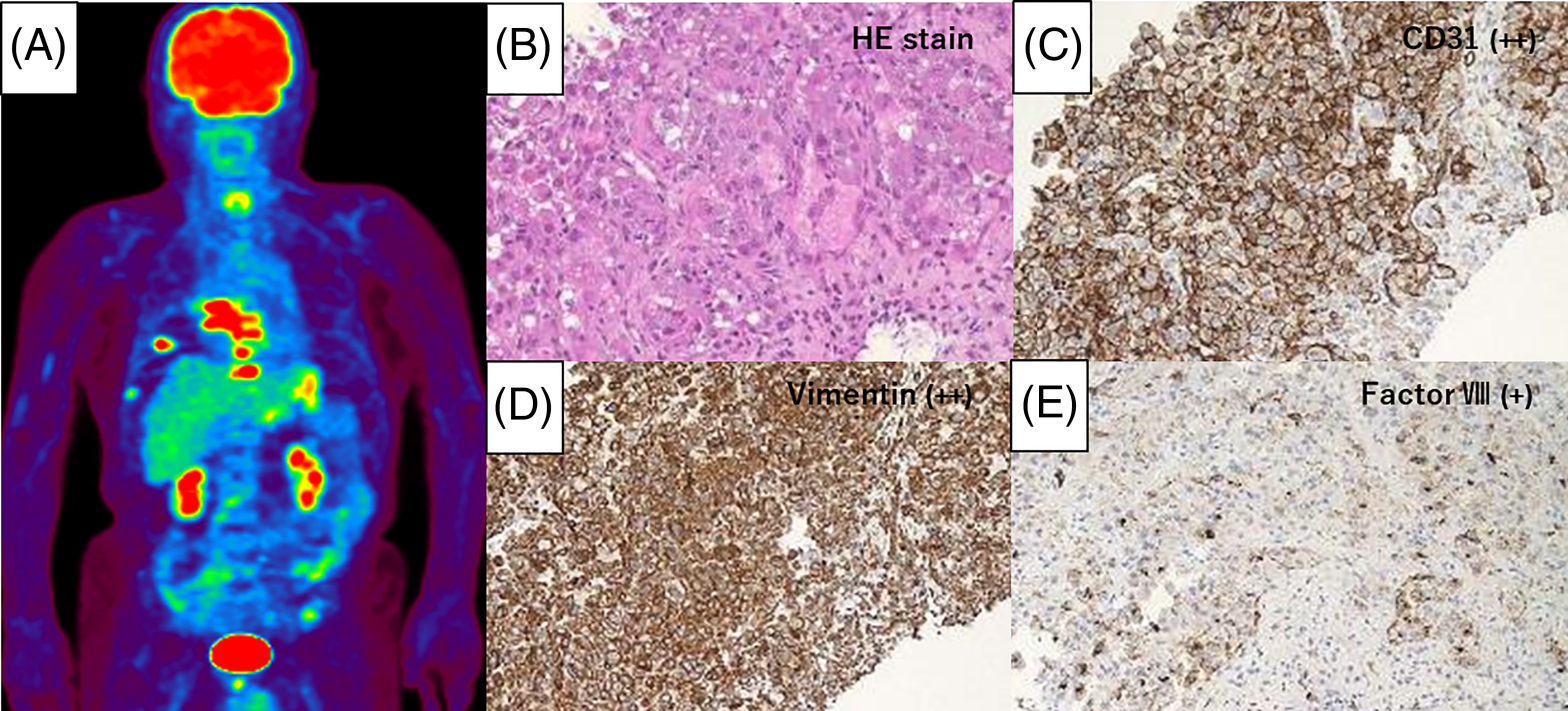
Positron‐emission tomography/computed tomography (PET‐CT) from 2 December 2017. (A) Uptake of 18F‐fluorodeoxyglucose (18‐FDG) is seen in the posterior protruding pleural thickening adjacent to the chronic empyema, the right seventh rib, sixth to ninth thoracic vertebral bodies, and the liver. (B) CT‐guided biopsy specimens showing malignant cells with increased nuclear size and glandular structure formation. Blood vessel‐like structures are also seen (haematoxylin and eosin stain). Immunohistochemical staining showing: (C) CD31 positivity on the cell membrane of malignant cells, (D) positivity for vimentin, and (E) slight positivity for factor VIII in the cytoplasm of malignant cells.

An 82‐year‐old man visiting our hospital for chronic tuberculous empyema had an annual follow‐up chest CT scan of the lesion. He had been treated for pulmonary tuberculosis with artificial pneumothorax at the age of 15 years. The CT scan at the end of August 2017 showed no change from the previous one. The patient first complained of right upper quadrant pain at the end of October 2017. The pain subsequently spread all over the right side of his chest, epigastric region, and back. He was admitted to our hospital for further examination on 11 November 2017. The contrast‐enhanced CT scan revealed irregular protrusion of the posterior pleural thickening adjacent to the chronic empyema, and the appearance of an abnormal right seventh costal mass, infiltrating the vertebral body (Fig. 1). He was diagnosed with angiosarcoma on 5 December 2017 based on the pathological findings of CT‐guided needle biopsy performed on 24 November 2017 (Fig. 2). His poor performance status made him ineligible for systemic chemotherapy. Instead, he received palliative radiotherapy (3 Gy/Fr, 30 Gy/10 Fr) for pain relief and prevention of spinal cord compression from multiple vertebral body metastases. After completion of radiotherapy, he was transferred to an inpatient hospice unit for further supportive care. The patient died of angiosarcoma on 15 February 2018.

## Discussion

Chronic pyothorax has been proven to have a definite association with malignant tumours. According to Aozasa et al., although angiosarcoma is a rare tumour, its occurrence in patients with pyothorax is 3600 times higher than that in the healthy population 2. Typical symptoms of pyothorax‐associated angiosarcoma include chest pain, haemoptysis, and palpable masses. Little is known about the difference between pyothorax‐associated angiosarcomas and other cancers in terms of symptoms and progression. In this case study, CT scans of the chest were obtained annually to monitor the chronic empyema, and to screen for cancers. The patient was diagnosed with angiosarcoma within 1 month of complaining of chest pain. The presence of bone and liver metastases at the time of diagnosis demonstrated the rapid growth of this cancer.

The primary treatment for angiosarcoma is surgical resection. However, as it grows rapidly, very few cases can undergo surgery. The mean duration of survival in pyothorax‐associated angiosarcoma is four months. Standard chemotherapy for angiosarcoma is uncertain due to poor prognosis.

By reviewing the literature, we identified nine cases of pyothorax‐associated angiosarcoma (Table 1). The duration between surgery for artificial pneumothorax and the diagnosis of angiosarcoma in those cases, ranged between 10 and 56 years 3, 4, 5, 6, 7, 8, 9. While five cases were diagnosed with angiosarcoma during autopsy, the four others were diagnosed while alive. In the cases diagnosed during life, several months elapsed between the first complaint of symptoms and the definite diagnosis.

**Table 1:** Nine cases of pyothorax‐associated angiosarcoma were identified on a review of the literature.

Case	Age	Gender	Duration of Op to Dx (years)	CT scan before FS	FS	Diagnostic method	Treatment	Duration of FS to Dx (months)	Duration of FS to death (months)	Author (Ref)	Year of publication
1	63	Male	About 40	UN	Chest pain	Autopsy	None	UN	UN	Hattori 3	2001
2	70	Female	13	None	Disorder of consciousness	Autopsy	None	2	2	Kimura et al. 4	2003
3	55	Male	About 30	Yes	Chest pain	Needle aspiration biopsy	Op, chemo	4	84 (7 years)	Katsura et al. 5	2004
4	85	Male	Over 10	UN	Haemoptysis	Surgical biopsy	Radiation	7	9	Katsura et al. 5	2004
5	72	Male	42	Yes	Back pain, haemoptysis	Autopsy	None	1	1	Katsura et al. 5	2004
6	76	Female	56	None	Haemoptysis	Autopsy	None	4	4	Kusano et al. 6	2004
7	76	Male	UN	None	Cough	Autopsy	None	5	5	Saitou and Niitsuma 7	2009
8	68	Male	UN	Yes	Haemoptysis	CT‐guided biopsy	Chemo	12	18	Bruixola et al. 8	2014
9	72	Male	Over 50	None	Chest pain	CT‐guided biopsy	None	UN	UN	Gorospe et al. 9	2017

CT, computed tomography; Dx, diagnosis; FS, the first symptom; Op, operation; Ref, reference; UN, unknown.

This is the very first report on angiosarcoma characterized by aggressive growth that could be observed on CT imaging performed at annual screenings. Unfortunately, yearly CT scans provided no benefits for our patient, as the rapid progression of his angiosarcoma hindered effective treatment options. Although the duration from the appearance of the first symptoms to the diagnosis of cancer was only one month, neither surgical treatment nor chemotherapy was possible. A more sensitive screening method should be developed for the early detection of angiosarcoma in patients with chronic empyema. Cumulative data from further cases are needed to validate our findings.

### Disclosure Statement

Appropriate written informed consent was obtained for publication of this case report and the accompanying images.
